# Effects of dietary *Clostridium autoethanogenum* protein on the growth, disease resistance, intestinal digestion, immunity and microbiota structure of *Litopenaeus vannamei* reared at different water salinities

**DOI:** 10.3389/fimmu.2022.1034994

**Published:** 2022-10-07

**Authors:** Jian Chen, Hongming Wang, Hang Yuan, Naijie Hu, Fangqi Zou, Chongyang Li, Lili Shi, Beiping Tan, Shuang Zhang

**Affiliations:** ^1^ College of Fisheries, Guangdong Ocean University, Zhanjiang, China; ^2^ Technology R&D Department, Beijing Shoulang Bio-Technology Co., Ltd., Beijing, China; ^3^ Key Laboratory of Aquatic, Livestock and Poultry Feed Science and Technology in South China, Ministry of Agriculture, Zhanjiang, China; ^4^ Aquatic Animal Nutrition and Feed Laboratory, Aquatic Animals Precision Nutrition and High Efficiency Feed Engineering Research Center of Guangdong Province, Zhanjiang, China

**Keywords:** *Litopenaeus vannamei*, *Clostridium autoethanogenum*, salinity, growth performance, intestinal microbiota

## Abstract

The shortage of fishmeal (FM) resources limits the healthy development of aquaculture. Developing new protein sources to replace FM in aquatic feeds is an effective measure to alleviate this situation. However, the application effect of new protein sources is greatly affected by water salinity, which is an important parameter of aquaculture. In this study, the growth, disease resistance, and intestinal digestion, immunity, and microbiota structure of *Litopenaeus vannamei* (initial weight: 0.38 ± 0.01 g) fed on *Clostridium autoethanogenum* protein (CAP) or not at three different water salinities (15 ‰, 30 ‰, and 45 ‰) were compared, aiming to explore the effects of dietary CAP on shrimp when suffering different salinity stresses. The results showed that the growth performance, feed utilization, and survival rate (SR) after pathogen challenge of *L. vannamei* could be significantly improved by dietary CAP when compared with the control at the same salinity and they were also significantly affected by salinity changes when *L. vannamei* was fed on the same protein source. With the increase in salinity, obvious upregulation was observed in the activities and gene expression of digestive enzymes both in *L. vannamei* fed on FM and CAP, with significantly higher levels in *L. vannamei* fed on CAP than in those fed on FM at the same salinity. Meanwhile, the expression levels of immune genes in the CAP group were significantly higher than those in the FM group at different salinities. The intestinal microbiota analysis showed that CAP could increase the relative abundance of beneficial bacteria and decrease the relative abundance of harmful bacteria in the intestine of *L. vannamei* at the phylum, family, and genus levels, and it was more affected by salinity changes when compared with FM. Besides, the changes in salinity and protein sources led to different changes in the intestinal microflora function of *L. vannamei*. In sum, this study indicated that CAP could improve the growth, disease resistance, digestive capacity, and intestinal microflora of *L. vannamei* with a much more intense immune response and enhance its ability to cope with salinity stress.

## 1 Introduction

Aquaculture can economically provide high-quality animal proteins for the global population (1). In the past 40 years, aquaculture production worldwide has increased rapidly to satisfy the increasing demand for animal protein consumption (2). Up to now, aquatic products have been considered the third-largest source of animal proteins (3). Rapidly increased aquaculture production results in a strong demand for fishmeal (FM), which is the indispensable source of high-quality protein in aquatic feeds (4). The shortage of FM has become one of the biggest problems facing the global aquatic feeds industry, which has seriously affected the healthy and sustainable development of the aquatic feeds industry (5). The threat in the future is that overfishing will lead to an ecological imbalance in the marine ecosystem because FM mainly comes from fishing (6). Thus, the development of suitable novel protein sources will meet the growing needs of the rapidly growing aquaculture industry (7).

*Litopenaeus vannamei* is the largest shrimp species in the world in consumption and cultivation. The annual production of *L. vannamei* in 2019 was 5.5 million tons (8). Generally, the protein requirements of *L. vannamei* are high and the best protein level for shrimp feed is 35% to 40% (9). FM is also the most common protein source for *L. vannamei* as for most aquatic animals, accounting for 30% of the feeds (10). Therefore, looking for substitutes for FM is of great significance for the healthy development of *L. vannamei* aquaculture. *L. vannamei* is a euryhaline shrimp species with a salinity tolerance range of 0.5–78 ‰ (11, 12). Salinity is a crucial environmental factor for aquatic animal reproduction, growth, development, and survival (13–15). In general, *L. vannamei* could effectively maintain osmotic pressure and ion regulation under different salinities to adapt to the environment (16). However, the survival, growth, and immunity varied according to the different salinities, which was mainly due to the energy digestibility changes (17–20). *L. vannamei* provides sufficient energy to effectively cope with changes in salinity mainly through food intake. If not, it uses its own body energy sources, resulting in slow growth, low survival rate, and so on (21). It has been reported that protein is a fundamental source of energy during salinity changes. *L. vannamei* reared in high salinity required high dietary protein than those reared in low saline waters (22, 23). This does not support sustainable aquaculture growth since high protein levels not only increase the cost of the feeds but also increase protein catabolism, which increases the organic load and environmental pollution (21). Therefore, it is essential to identify cheap protein energy sources to spare dietary protein in the shrimp culture.

At present, the development and application of novel protein sources in shrimp feeds mainly focus on plant protein, animal protein, and single-cell protein (SCP) sources (24). However, the use of plant protein is limited due to its unbalanced amino acid composition, the presence of anti-nutritional factors, and poor taste. At the same time, the nutritional content of animal protein sources usually varies with the season or product batch, and animal protein’s biological safety must also be considered. SCP, also known as microbial protein, is not only rich in protein and amino acids but also in vitamins, minerals, nucleotides, and immune polysaccharides and has gotten a lot more favour (25). *Clostridium autoethanogenum* protein (CAP) is a by-product of *Clostridium autoethanogenum* fermentation to produce ethanol. *Clostridium autoethanogenum* can produce ethanol and CAP by using CO produced by industrial tail gas as a carbon source and ammonia water as a nitrogen source. As a high-quality SCP, the protein content of CAP can be as high as 80%. Besides, CAP is rich in essential amino acids, easy for animals to digest and absorb, and contains no anti-nutritional factors. CAP has been used in several aquaculture species, including *Acanthopagrus schlegelii*, *Micropterus salmoides*, *Cyprinus carpio* var. *Jian*, and *L. vannamei*, with the effect of improving growth performance, feed utilization, anti-oxidation, intestinal health, and immune response (26–31). In a basal diet containing 560 g/kg FM, CAP could substitute 30% FM without adverse effects on growth, intestinal histology, and immunity, while higher FM substitution decreased the growth and flesh quality of *L. vannamei (* 27, 32). However, the application effect of CAP on *L. vannamei* at different salinities has not been investigated up to now.

In this study, the ability of *L. vannamei* to utilize CAP at different salinities was investigated from the aspects of growth performance, disease resistance, intestinal digestive capacity, immunity, and microbiota structure, which could provide a theoretical reference for the application of *C. autoethanogenum* as a new protein source in aquatic feeds.

## 2 Materials and methods

### 2.1 Diet preparation

Two isoproteic and isolipidic diets were made as shown in **Table 1**. The control diet was designed using FM (589 g/kg) as the only protein source. Under the premise of using 150 g/kg FM to meet the basic requirement for normal growth (10), 354g/kg CAP was used as the only protein source to replace FM in the experimental diet. All raw materials were crushed and screened through an 80-mesh screen. After the raw materials were mixed by a step-by-step expanding method, they were fully mixed by a V-type mixer (JS-14S, Zhejiang Chint Electrics Co., Ltd., Zhejiang, China). Fish oil, corn oil, and soybean lecithin were added and mixed again, followed by adding some water to the mixture, and then extruded using a twin-screw extruder (M-256, South China University of Technology, Guangzhou, China). The feed pellets were baked at 75°C for 20 min and air-dried naturally, and then stored in the refrigerator at 20°C. CAP was provided by Beijing Shoulang Bio-technology Co., Ltd., Beijing, China. The crude protein, crude lipid, crude ash, and moisture of CAP were 84.21%, 0.19%, 3.27%, and 7.14%, respectively.

**Table 1:** The formula and proximate composition of the diet (dry matter/**%**).

Ingredients (%)	Groups	
	FM	CAP
Brown fish meal	58.90	15.00
CAP	0.00	35.40
Corn starch	20.00	20.00
Fish oil	0.36	2.52
Corn oil	0.36	2.52
Soyabean lecithin	1.00	1.00
Vitamin and mineral premix^a^	1.20	1.20
Choline chloride	0.50	0.50
Ethoxyquin	0.05	0.05
Attractant^b^	0.10	0.10
Ca(H_2_PO_4_)_2_	1.20	1.20
Vitamin C	0.05	0.05
Cellulose microcrystalline	16.28	20.45
Total	100.00	100.00
**Proximate composition (%)**		
Crude protein^c^	41.39	40.85
Crude lipids^c^	7.53	7.66
Ash	11.56	5.16
Moisture	7.32	7.64

^a^Vitamin and Mineral Premix (kg^−1^ of diet) includes the following contents: thiamine, 5 mg; riboflavin, 10 mg; vitamin A, 5,000 IU; vitamin D3, 1,000 IU; vitamin E, 40 mg; menadione, 10 mg; pyridoxine, 10 mg; biotin, 0.1 mg; cyanocobalamin, 0.02 mg; calcium pantothenate, 20 mg; folic acid, 1 mg; niacin, 40 mg; vitamin C, 150 mg; FeSO_4_·H_2_O, 303 mg; KIO_3_, 1.3 mg; Cu_2_(OH)_3_Cl, 5 mg; ZnSO_4_·H_2_O, 138 mg; MnSO_4_·H_2_O, 36 mg; Na_2_SeO_3_, 0.6 mg; CoCl_2_·6H_2_O, 0.8 mg.

^b^The attractant is betaine.

^c^Crude protein and crude lipid contents were measured value.

### 2.2 Collection and acclimatization of the trialled *L. vannamei*

The *L. vannamei* larvae were provided by Zhanjiang Yuehai Aquatic Fry Co., Ltd. (Zhanjiang, China). 240 *L. vannamei* (initial weight of 0.38 ± 0.01 g) were randomly divided into two equal groups and fed on FM or CAP diets. The FM or CAP group was equally divided into three subgroups at three salinities of low salinity (15 ‰), medium salinity (30 ‰), and high salinity (45 ‰), which were set as previous studies (33, 34). Three biological replicates were set for each subgroup with 40 individuals placed in 300-litre fiberglass tanks. *L. vannamei* were further adapted to low and high salinity. Artificial seawater salt (Jiangxi Yantong Technology Co., Ltd., Jiangxi, China) was used to gradually increase salinity to 45‰ for high salinity, and freshwater was used to gradually decrease salinity to 15‰ for low salinity from the original medium salinity of 30‰, being changed by 2‰ per day. Thus, there were six treatments with three replicates per treatment, that is FM15‰, FM30‰, FM45‰, CAP15‰, CAP30‰, and CAP45‰ groups, respectively. *L. vannamei* were fed four times daily at the following times: 7:00 am, 11:00 am, 17:00 pm, and 21:00 pm. At the beginning of the experiment, *L. vannamei* were fed on an amount of feeds equivalent to 10% of their body weight. 0.1 g of feed was added to the amount of feed per tank per day if the shrimp finished eating within 30 minutes. One-third of the water was replaced each day by water with pre-adjusted salinity from a reservoir. The temperature, ammonia nitrogen, dissolved oxygen, and pH were monitored daily and maintained between 27–30°C, <0.05 mg/L, >6.0 mg/L, and 7.7–8.0, respectively.

### 2.3 Sample collection

After 24-hour starvation at the end of 8 weeks of feeding, the shrimps were counted and weighed to measure survival rate (SR) and the overall body weight indicators, including the final body weight (FW), weight gain rate (WGR), specific growth rate (SGR), and protein efficiency ratio (PER). Intestines from nine shrimps were randomly collected from each tank into three samples, to analyze the intestinal digestive enzyme activity, gene expression, and microbiome structure. After being placed in liquid nitrogen for rapid freezing, samples were transferred to −80°C storage for subsequent analysis.

### 2.4 Growth performance analysis

Based on the recorded data, the indices for the assessment of growth performance, including SR, WGR, SGR, FCR, and PER, were calculated as follows:


$$\text{SR(\%})=\frac{\text{Final shrimp number}}{\text{Initial shrimp number}}\times 100$$

$${\text{SGR (\% d}}^{-1})=\frac{\left[\text{Ln (Final body weight) }-\text{ Ln (Initial body weight})\right]}{\text{Days}}\times 100$$

$$\text{WGR (\%})=\frac{\text{(Final body weight }-\text{ Initial body weight)}}{\text{Initial body weight}}\times 100$$

$$\text{FCR}=\frac{\text{Feed intake}}{\text{(Final body weight }-\text{ Initial body weight)}}$$

$$\text{PER (\%})\;=\frac{\text{(Final body weight }-\text{ Initial total weight)}}{\text{Protein intake}}\times 100$$

### 2.5 Challenge tests

*Vibrio parahaemolyticus* were prepared as in our previous studies (35, 36). The *V. parahaemolyticus* cells were centrifuged (5000 g) for 10 min at 4°C and then resuspended by 1 × PBS as an inoculum about 1 × 10^5^ colony-forming units (CFU)·µL^−1^. After sample collection, a total of 30 *L. vannamei* from each group were chosen to perform a challenge test with *V. parahaemolyticus* at a dose of 10^7^ CFU/g shrimp. The survival rate was recorded every 4 hours. The differences between the two groups were analyzed by the GraphPad Prism software using the Mantel-Cox (log-rank χ2 test) method.

### 2.6 Detection of intestinal digestive enzyme activities

50 mg of intestinal tissue was added into 0.01 mol/L PBS (PH = 7.2–7.4) to prepare a homogenate at a proportion of 10% (Tissue: PBS=1: 9). About 20 minutes after centrifugation (2000–3000 rpm/min), the supernatant was collected and examined. The activities of three digestive enzymes, including trypsin, amylase, and lipase, were determined using the enzyme-linked immunosorbent assay (ELISA) kit ml036384, ml036449, and ml036371 from Shanghai Enzyme-linked Biotechnology Co., Ltd., respectively. The relevant operation was carried out strictly according to the manual.

### 2.7 Gene expression analysis of intestinal digestive enzymes

The total RNA of the intestine was extracted by TransZol Up Plus RNA kits (TransGen, China), and the RNA concentration was determined by Spectrophotometric analysis (Nanodrop 2000). The cDNA was reverse-transcribed from total RNA by Evo M-MLV RT kit with gDNA Clean for qPCR II (Accurate Biotechnology Hunan Co., Ltd, China). The gene expressions of trypsin, amylase, lipase, superoxide dismutase (SOD), lipopolysaccharide (LPS) and beta-1,3-glucan binding protein (LGBP), prophenoloxidase (PPO), phenoloxidase (PO), Crustin (CRU), anti-lipopolysaccharide factor (ALF), penaeidin (PEN), and lysozyme (LYZ) were assessed by the Roche Light Cycler480 thermal cycler (Roche Applied Science, Germany) using the SYBR^®^ Green Premix Pro Taq HS qPCR Kit II (Accurate Biotechnology Hunan Co., Ltd, China). For each target gene, specific primers were designed by Primer 5.0 software according to the known sequences in the NCBI database (**Table 2**). The results of real-time qPCR were analyzed by the 2^−ΔΔCT^ method (37) using elongation factor 1α (EF1α) as a reference gene. Three independent biological replicates were performed for each sample.

**Table 2:** PCR primers used in this study.

Gene names	Primers	GenBank no.	Sequences (5’-3’)
EF1α	EF1α-F	XM027373349.1	GTATTGGAACAGTGCCCGTG
	EF1α-R		TCACCAGGGACAGCCTCAGTA
Lipase	Lipase-F	XM02373566.1	TCTCCCACTTCAATCGTCA
	Lipase-R		ATGCTTGGAATCGCTCTG
Trypsin	Trypsin-F	JQ277721.1	CTTCCGCCGTGGTCTCAA
	Trypsin-R		TCTGCTCGGTGCCCTCAT
Amylase	Amylase-F	XM027369804.1	GTTCCTTACTCCGCTTTCG
	Amylase-R		CGTAGTCAGTGCCTTGGTTCA
SOD	SOD-F	DQ005531.1	CTTTGCCACCCCTCAAGTATG
	SOD-R		TGCCTCCGCCTCAACCA
GBP	GBP-F	AY723297.1	TACGGAGGAACGACGCTGC
	GBP-R		AAATCATCGGCGAAGGAGC
PPO	PPO-F	AY723296.1	AACTCCATTCCGTCCGTCTG
	PPO-R		CGGCTTCGCTCTGGTTAGG
PO	PO-F	XM027381766.1	AAGCCAGGCAGCAACCAC
	PO-R		CAGAAGTTGAAACCCGTGGC
CRU	CRU-F	AF430071.1	GTAGGTGTTGGTGGTGGTTTC
	CRU-R		CTCGCAGCAGTAGGCTTGAC
ALF	ALF-F	EW713395	TTACTTCAATGGCAGGATGTGG
	ALF-R		GTCCTCCGTGATGAGATTACTCTG
PEN	PEN-F	DQ206401.1	GACGGAGAAGACAATGGAAACC
	PEN-R		ATCTTTAGCGATGGATAGACGAA
LYZ	LYZ-F	AF425673.1	TATTCTGCCTGGGTGGCTTAC
	LYZ-R		CAGAGTTGGAACCGTGAGACC

EF1α, elongation factor 1-alpha, SOD, superoxide dismutase, GBP, beta-1,3-glucan binding protein, PPO, prophenoloxidase, PO, phenoloxidase, CRU, small cysteine and glycine repeat-containing protein, ALF, anti-lipopolysaccharide factor, PEN, Penaeidin, LYZ, lysozyme.

### 2.8 Intestinal microbial analysis

The genomic DNA of the microorganisms was extracted from intestinal samples following the manufacturer’s instructions using HiPure Soil DNA Kits (Magen, Guangzhou, China). The V3-V4 region of the 16S rDNA gene was amplified using primers 341F: CCTACGGGNGGCWGCAG; 806R: GGACTACHVGGGTATCTAAT. The PCR program was conducted at an initial denaturation step at 95°C for 5 min, followed by 30 cycles at 95°C for 1 min, 60°C for 1 min, 72°C for 1 min, and a final extension at 72°C for 7 min. PCR reactions were performed in a triplicate 50-μL mixture containing 10 μL of 5 × Q5^@^ Reaction Buffer, 10 μL of 5 × Q5^@^ High GC Enhancer, 1.5 μL of 2.5 mM dNTPs, 1.5 μL of each primer (10 μM), 0.2 μL of Q5@ High-Fidelity DNA Polymerase, and 50 ng of template DNA. The related PCR reagents were from New England Biolabs, USA. The amplified products were purified by the AxyPrep DNA gel extraction kit (Axygen Biosciences, Union City, CA, USA). Subsequently, amplicons were pooled into equimolar concentrations and sequenced by Guangzhou Genedenovo Biotechnology Co., Ltd., using a Hiseq2500 PE250 machine (Illumina, USA). The raw data were deposited in the NCBI GenBank (http://www.ncbi.nlm.nih.gov/genbank/).

To obtain high-quality clean reads, FASTP (38) was used to further filter the raw reads and the noise sequence of the raw tags under specific filtering conditions to obtain high-quality clean tags. The clean tags were clustered into operational taxonomic units (OTUs) of ≥ 97% similarity using UPARSE pipeline (38). The representative OTU sequences were classified using the RDP classifier (39) based on the SILVA database (40), with a confidence threshold value of 0.8. Alpha diversity indexes, OTUs, Chao 1, ace, Shannon, Simpson, and Goods coverage were calculated using QIIME (41, 42). Beta diversity indexes and principal coordinates analysis (PCoA) of bray-curtis distances were generated in the R project Vegan package (43). The KEGG pathway of OTUs was analyzed by Tax4Fun (42).

### 2.9 Statistical analysis

The results were expressed as mean ± standard deviation (mean ± SD), and two-way analysis of variance (ANOVA) was used to test the significance using SPSS 20.0 statistical software. Tukey’s multiple comparison method was further used if there were significant differences. For the challenge test, the survival rate was calculated using Log-rank Kaplan-Meier analysis by GraphPad Prism. The differences between all the test results were considered significant at *P*< 0.05 and highly significant at *P*< 0.01.

## 3 Results

### 3.1 Growth performance and feed utilization

Analysis of variance analysis (**Table 3**) showed that the protein source and salinity significantly affected the WGR, SGR, FCR, and PER but not the SR of *L. vannamei*. Besides, the interaction of protein source and salinity had a significant effect on all these five indices of *L. vannamei* (*P<* 0.05).

**Table 3:** Effects of protein sources and salinity on growth of *L. vannamei*.

Items		SR/%	WGR/%	SGR/(%/d)	FCR	PER (%)
Protein source	Salinity/‰				
**FM**	**15**	88.75 ± 1.77	1757.77 ± 27.12^b^	5.22 ± 0.03^b^	1.41 ± 0.12^B^	1.76 ± 0.16^A^
	**30**	90.00 ± 2.50	1784.13 ± 36.33^Ab^	5.24 ± 0.03^Ab^	1.49 ± 0.04^A^	1.66 ± 0.05^B^
	**45**	95.83 ± 1.44	1588.92 ± 23.43^Aa^	5.05 ± 0.12^Aa^	1.39 ± 0.10^A^	1.78 ± 0.13^B^
**CAP**	**15**	92.50 ± 2.50	1843.40 ± 35.86^a^	5.30 ± 0.03^a^	1.04 ± 0.05^Aa^	2.36 ± 0.10^Bc^
	**30**	94.17 ± 1.44	1983.43 ± 59.56^Bb^	5.42 ± 0.05^Bb^	1.98 ± 0.09^Bb^	1.25 ± 0.06^Ab^
	**45**	91.17 ± 3.75	1790.54 ± 65.73^Ba^	5.25 ± 0.02^Ba^	3.69 ± 0.09^Bc^	0.67 ± 0.02^Aa^
***P*-value of two-way ANOVA**						
**Salinity**		0.1981	<0.0001	<0.0001	<0.0001	<0.0001
**Protein source**		0.3819	<0.0001	<0.0001	<0.0001	<0.0001
**Interaction**		0.0161	0.0279	0.0199	<0.0001	<0.0001

Values with different capital letter superscripts indicated significant differences between different protein sources at the same salinity (P<0.05). Values with different small letter superscripts indicated significant difference among different salinities when the protein source was the same (P<0.05). The same as below.

The protein source changes led to significant changes in the WGR, SGR, FCR, and PER of *L. vannamei* at the same salinity. At the same salinity, a change in protein source resulted in a significant difference in the growth performance and feed utilization of *L. vannamei* between the FM and the CAP groups. At 15‰ salinity, the FCR of *L. vannamei* in the CAP group was significantly lower than that in the FM group, while the PER was significantly higher than that in the FM group (*P*< 0.05). The WGR, SGR, and FCR of *L. vannamei* in the CAP group at 30‰ and 45‰ salinity were significantly higher than those in the FM group, while the PER was significantly lower than that in the FM group (*P*< 0.05).

Besides, the alteration of salinity led to different changes in the growth performance and feed utilization of *L. vannamei* in the FM and the CAP groups. The WGR and SGR of *L. vannamei* were significantly different at different salinities both in the FM and the CAP groups. However, only the FCR and PER of *L. vannamei* in the CAP group were significantly different at different salinities. In the FM group, the WGR and SGR of *L. vannamei* at 45‰ salinity were significantly decreased when compared with those at 15‰ and 30‰ salinities. In the CAP group, the WGR and SGR of *L. vannamei* were highest at 30‰ salinity, with a significantly higher level than those at 15‰ and 45‰ salinities. With the increase in salinity, the FCR of *L. vannamei* increased significantly while the PER of *L. vannamei* decreased significantly (*P<* 0.05).

### 3.2 Survival rates of *L. vannamei* after *V. parahaemolyticus* infection

**Figure 1:**
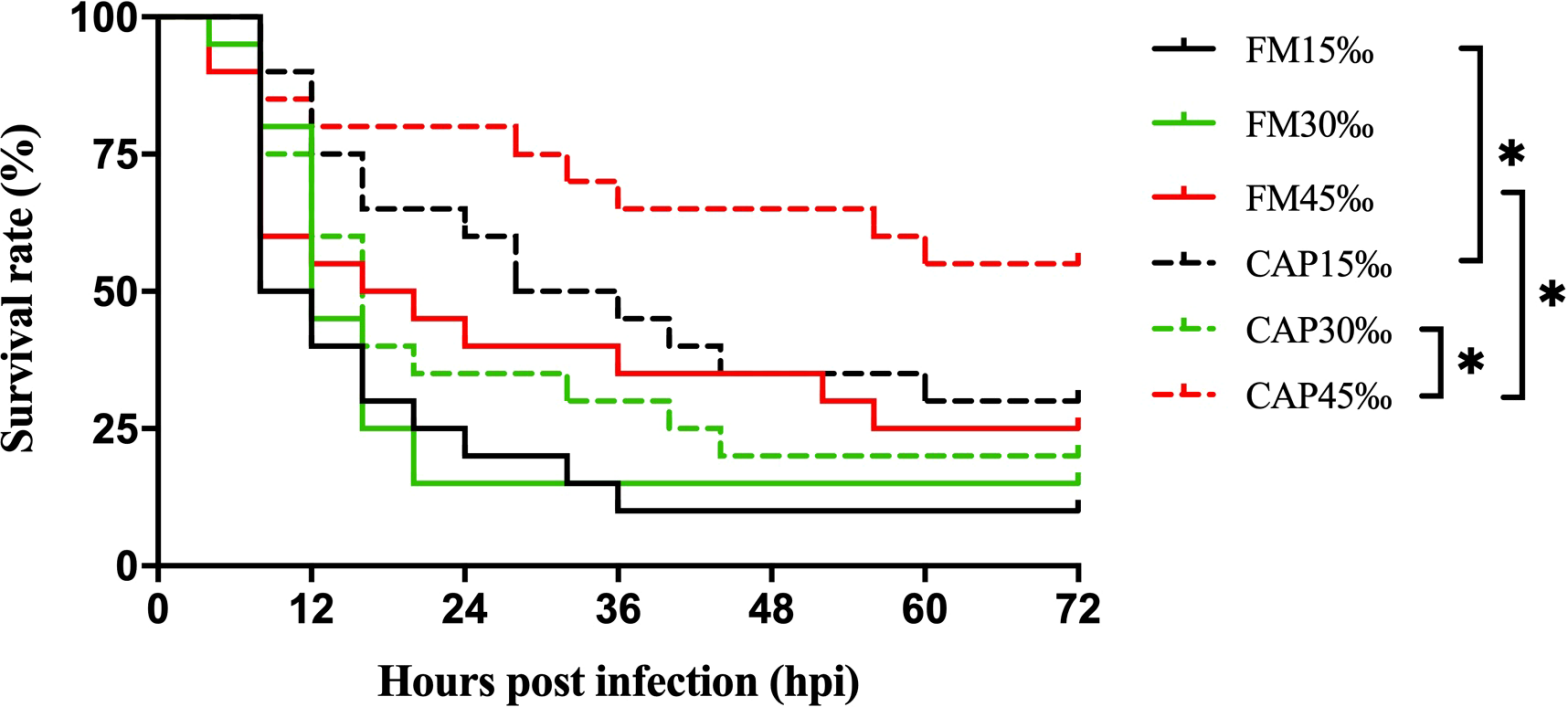
Survival rates of L. vannamei after V. parahaemolyticus infection. Differences in survival levels between treatments were analyzed by Kaplan-Meier plot (log-rank *χ* ^2^ test). Significant differences in survival rate were marked with asterisks, * indicates *P*< 0.05.

The survival rates of *L. vannamei* after *V. parahaemolyticus* infection were not significantly different among the FM group at the three different salinities. In the CAP group, the survival rate of *L. vannamei* at 45‰ salinity after *V. parahaemolyticus* infection was highest and significantly higher than that at 30‰ salinity (**Figure 1**). Under the condition of the same salinity, the survival rate of *L. vannamei* in the CAP group after *V. parahaemolyticus* infection was higher than that in the FM group, with significantly higher levels at 15‰ and 45‰ salinities (*P*< 0.05).

### 3.3 Digestive enzyme activities in the intestine

**Figure 2:**
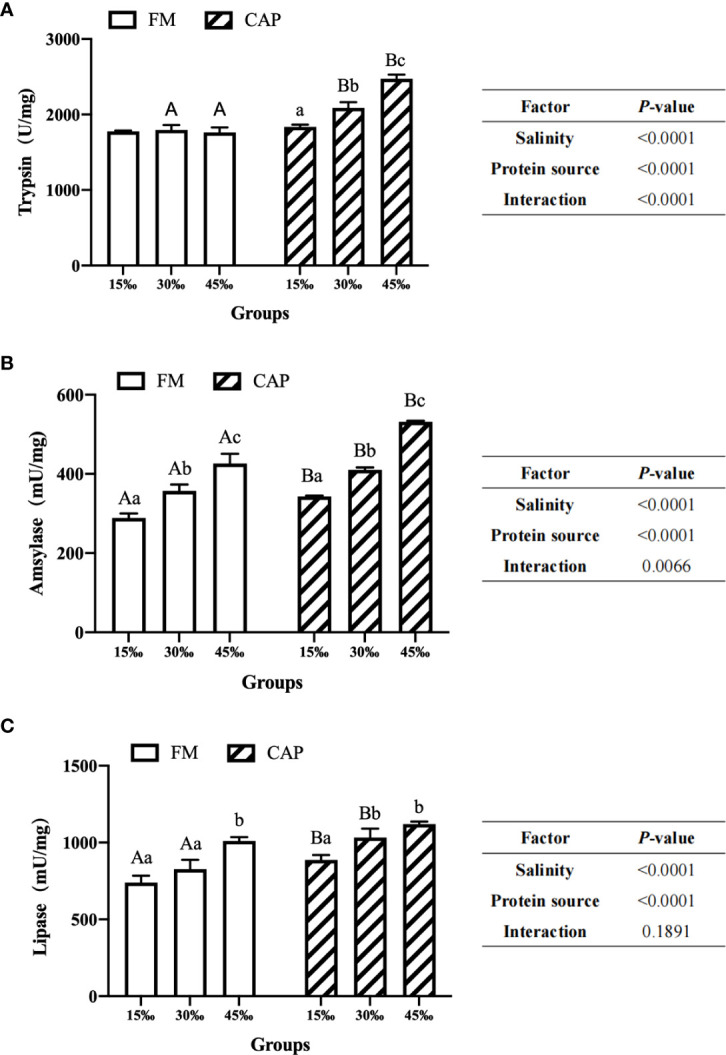
Effects of protein sources and salinity on intestinal trypsin **(A)**, amylase **(B)** and lipase **(C)** activities in L. vannamei. Under the same salinity, values with different capital letter superscripts mean significant difference among different protein source (*P*<0.05); under the protein sources, values with different small letter superscripts mean significant difference between different salinity (*P<* 0.05).

As shown in **Figure 2**, under the condition of the same salinity, the intestinal amylase and lipase activities of *L. vannamei* in the CAP group were significantly higher than those in the FM group at 15‰ salinity (*P*< 0.05). At 30‰ salinity, the activities of all the three intestinal digestive enzymes of *L. vannamei* in the CAP group were significantly higher than those in the FM group (*P*< 0.05). At 45‰ salinity, the activities of trypsin and amylase but not that of lipase in the CAP group were significantly increased when compared with those in the FM group (*P*< 0.05).

The activities of three intestinal digestive enzymes, including trypsin, amylase and lipase, were significantly up-regulated with the increase in the salinity both in the FM and the CAP groups, with the only exception being that the trypsin activities in the FM group at different salinities were not significantly changed. In the FM group, amylase activities in the intestine of *L. vannamei* at three different salinities were significantly different, with the highest level at 45‰ salinity. The intestinal lipase activity of *L. vannamei* at 45‰ salinity was significantly higher than those at 15‰ and 30‰ salinities. However, there was no significant difference between the activities of lipase in the intestine of *L. vannamei* at 15‰ and 30‰ salinities. In the CAP group, both the intestinal trypsin and amylase activities of *L. vannamei* were significantly induced by the increase in salinity, reaching the peak at 45‰ salinity. The activities of intestinal lipase in *L. vannamei* at 30‰ and 45‰ salinities were not significantly different but both of them were significantly higher than that at 15‰ salinity.

Two-way ANOVA showed that the protein source and salinity significantly affected the activities of trypsin, amylase, and lipase in the intestine of *L. vannamei*. The interaction of protein source and salinity had a significant effect on the activities of trypsin and amylase but not on that of lipase.

### 3.4 The gene expression of intestinal digestive enzymes

**Figure 3:**
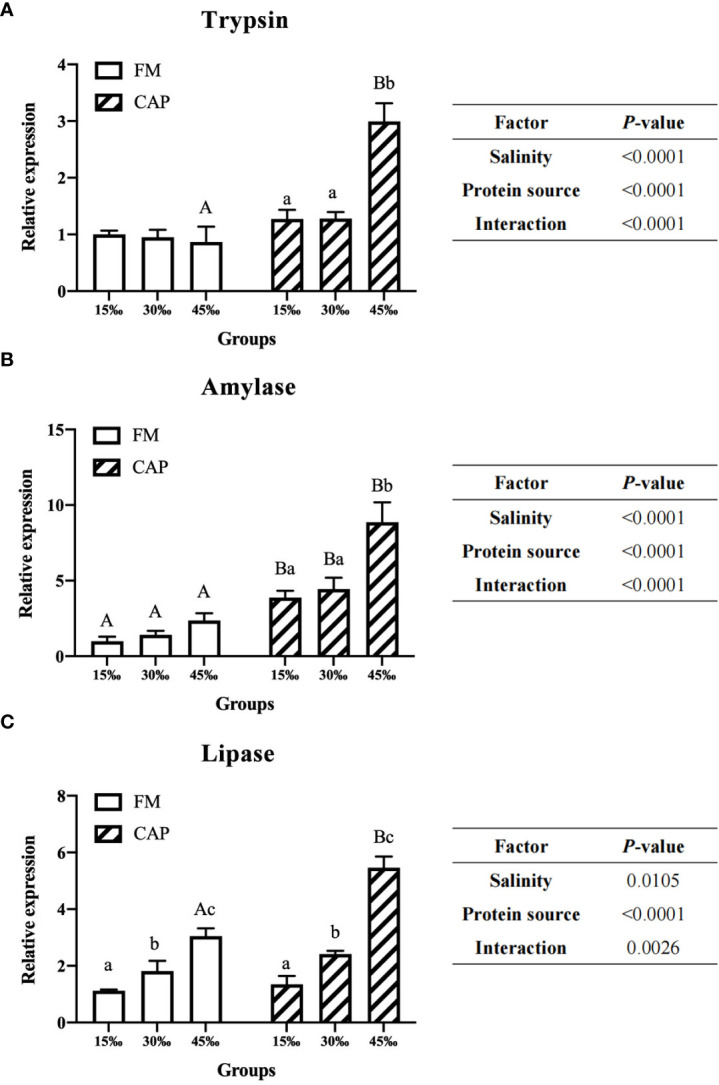
Effects of protein source and salinity on the gene expression of intestinal degistive enzymes in L. vannamei. The detection of gene expression were performed in triplicate for each sample. Expression values were normalized to those of EF1α using the Livak (2^-ΔΔCt^) method and the data were provided as the means ± SD of triplicate assays. Under the same salinity, values with different capital letter superscripts mean significant difference among different protein source (*P* < 0.05). Under the protein sources, values with different small letter superscripts mean significant difference between different salinity (*P* < 0.05). **(A)** Trypsin gene expression, **(B)** Amylase gene expression, **(C)** Lipase gene expression.

When compared with those in the FM group (**Figure 3**), the expression levels of intestinal Trypsin and Lipase genes of *L. vannamei* in the CAP group were significantly higher only at 45‰ salinity, while the intestinal Amylase expression levels were significantly increased at all the studied salinities (*P*< 0.05). The gene expression levels of Lipase but not those of Trypsin and Amylase in the intestine of *L. vannamei* were significantly raised by the increase in salinity in the FM group. However, there was no significant difference in the gene expression of Trypsin and Amylase at different salinities. In the CAP group, the intestinal Lipase expression levels of *L. vannamei* at the three different salinities were significantly different from each other. Both the intestinal Trypsin and Amylase expression at 45‰ salinity were significantly higher than those at 15‰ and 30‰ salinities; there was no significant difference between the FM group and the CAP group. Two-way ANOVA showed that the protein source, salinity, and their interaction had a significant effect on the expression of Trypsin, Amylase, and Lipase in the intestine of *L. vannamei*.

### 3.5 The expression of immune genes in the intestine

**Figure 4:**
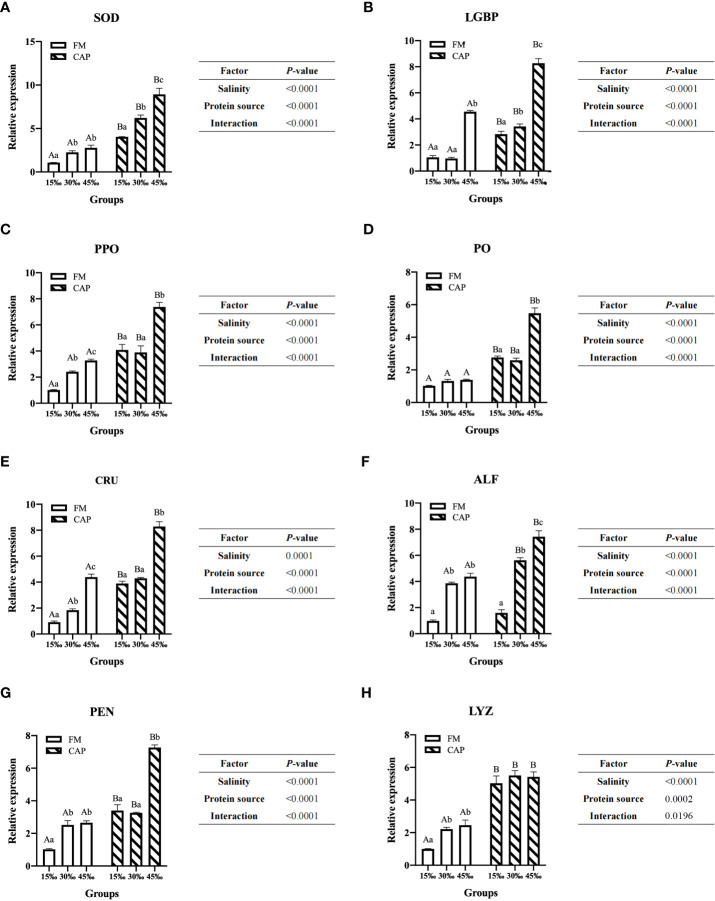
Effects of protein source and salinity on intestinal immune gene expression in L. vannamei. The detection of gene expression were performed in triplicate for each sample. Expression values were normalized to those of EF1α using the Livak (2^-ΔΔCt^) method and the data were provided as the means ± SD of triplicate assays. Under the same salinity, values with different capital letter superscripts mean significant difference among different protein source (P < 0.05) The expression of immune genes in the intestine. Under the protein sources, values with different small letter superscripts mean significant difference between different salinity (*P* < 0.05). **(A)** SOD gene expression, **(B)** LGBP gene expression, **(C)** PPO gene expression, **(D)** PO gene expression, **(E)** CRU gene expression, **(F)** ALF gene expression, **(G)** PEN gene expression, **(H)** LYZ gene expression.

Compared with that in the FM group (**Figure 4**), the expression of immune genes in the intestine of *L. vannamei* in the CAP group was significantly increased at different salinities (*P*<0.05), with the only exception being that ALF expression was not significantly different between the FM and the CAP groups at 15‰ salinity. In the FM and the CAP groups, a change in salinity led to a significantly increased expression of the detected immune genes except for PO in the FM group and LYZ in the CAP group. In the FM group, the expression levels of PPO and CRU genes in the intestine of *L. vannamei* at different salinities were significantly different, with the highest level at 45‰ salinity. The LGBP expression at 45‰ salinity was significantly higher than that at 15‰ and 30‰ salinities (*P*<0.05), and the LGBP expressions at 15‰ and 30‰ salinities were not significantly different (*P*>0.05). The expression of SOD, ALF, PEN, and LYZ genes at 30‰ and 45‰ salinities were significantly higher than those at 15‰ salinity, and their expression levels at 30‰ and 45‰ salinity were not significantly different (*P*>0.05). In the CAP group, the gene expression levels of SOD and ALF in the intestine of *L. vannamei* were significantly different at different salinities, with the highest level at 45‰ salinity. The expressions of intestinal LGBP, PPO, PO, CRU, and PEN at 45‰ salinity were significantly higher than those at 15‰ and 30‰ salinities, but their expressions at 15‰ and 30‰ salinities were not significantly different. Two-way ANOVA showed that protein source, salinity, and their interactions had significant effects on the expressions of all the eight detected intestinal immune genes in *L. vannamei.*

### 3.6 Intestinal microbiota analysis

#### 3.6.1 Richness and diversity analysis

**Figure 5:**
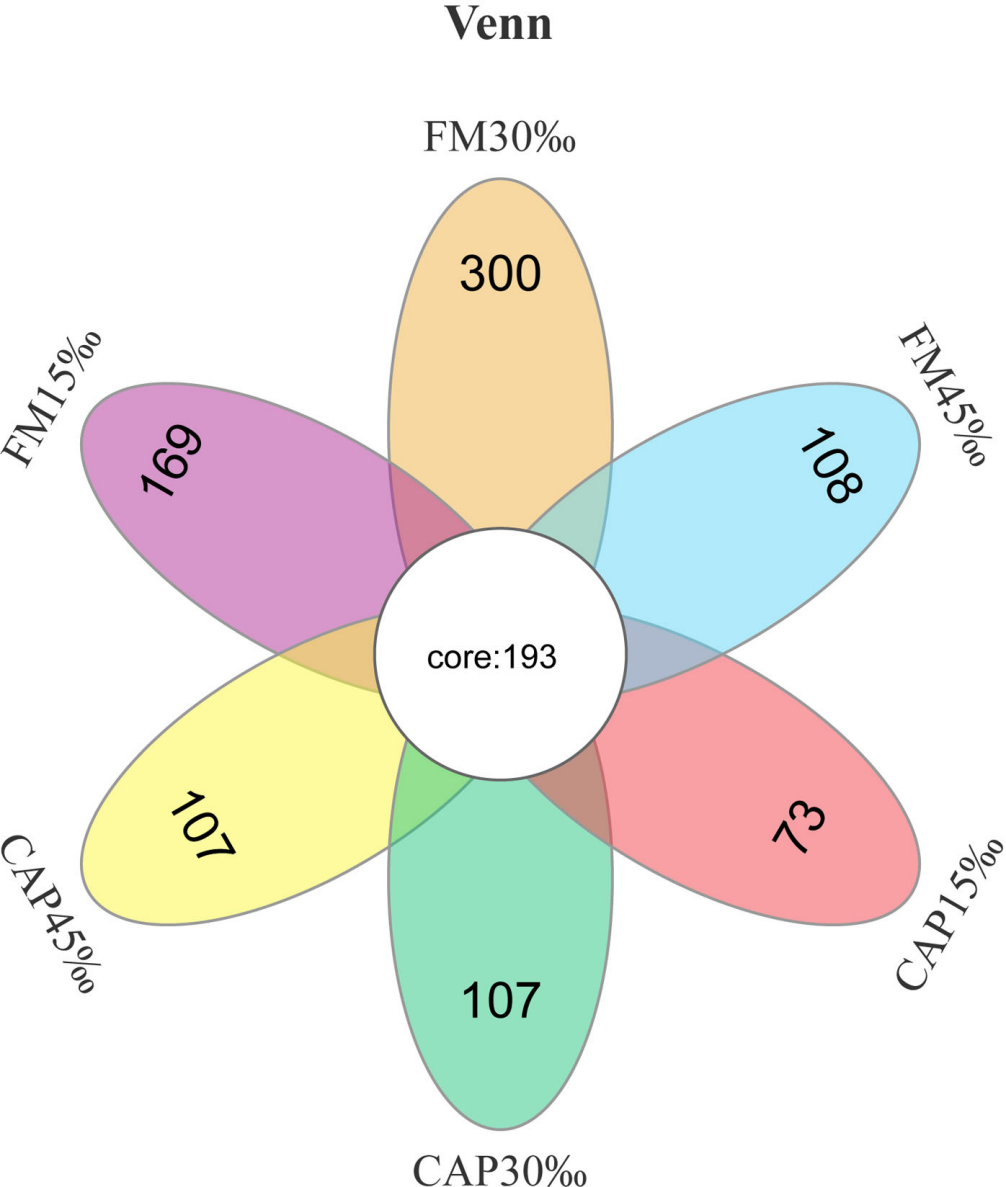
Venn diagram of shared and unique OTUs of intestinal microbiota in L. vannamei.

The raw data of intestinal microbiota analysis in this study have been deposited in the SRA database with the accession number PRJNA870236. As shown in **Figure 5**, there were 193 core operational taxonomic units (OTUs) among all the tested groups according to Venn diagram analysis. In contrast, 169, 300, 108, 73, 107, and 107 OTUs were unique to FM15‰, FM30‰, FM45‰, CAP15‰, CAP30‰, and CAP45‰ groups, respectively. Clearly, the proportions of shared OTUs within each group were 52.31%, 39.15%, 64.12%, 72.56%, 64.33%, and 64.33%, respectively.

**Figure 6:**
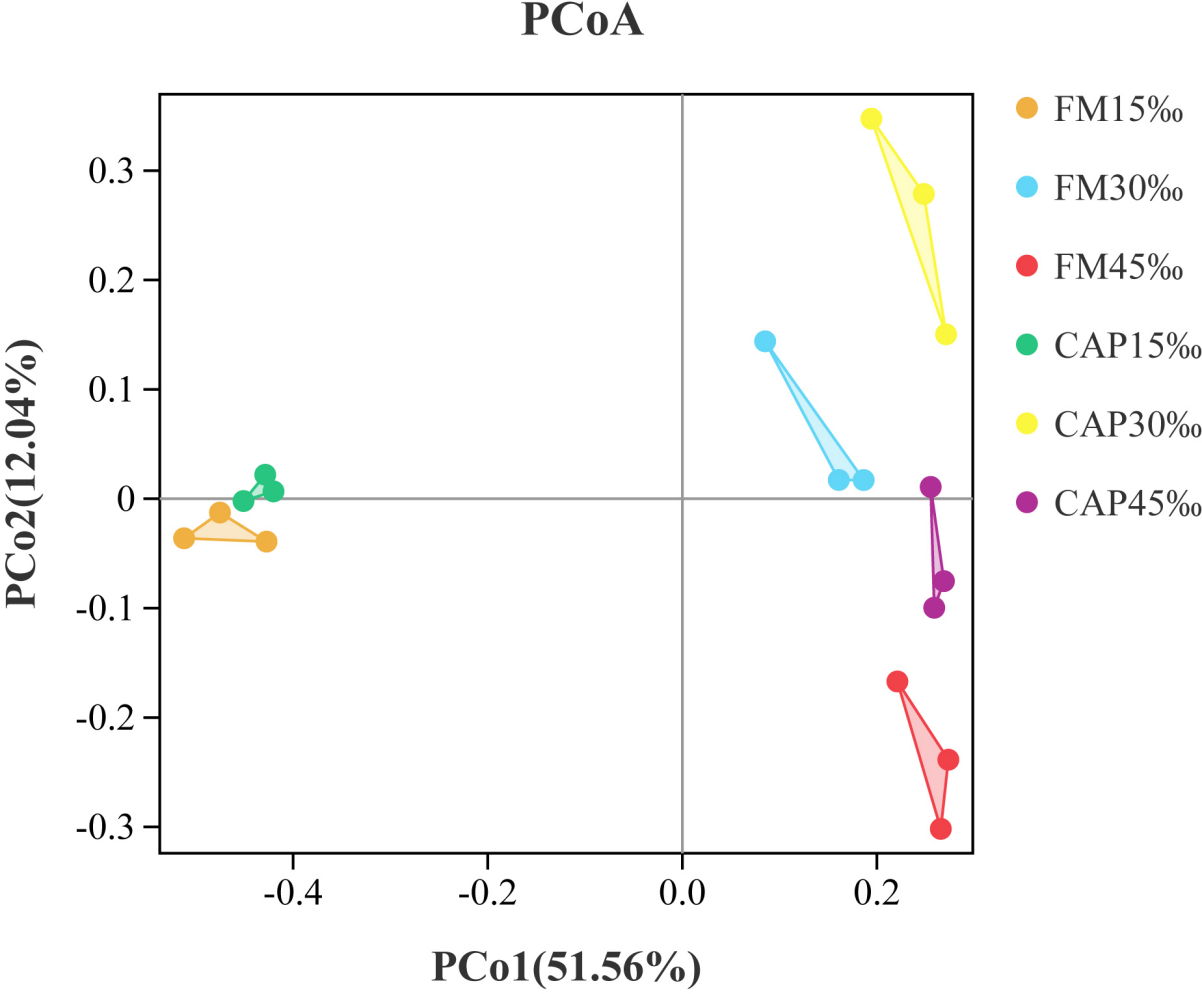
Principal coordinates analysis (PCoA) based on Bray analysis of intestinal microbiota in L. vannamei.

Alpha indices, including Good’s coverage, observed species (Sobs), Chao1, abundance-based coverage estimator (ACE), Shannon, and Simpson, were evaluated to investigate the significant differences in the diversity and richness of microbiota in the intestine among different treatments (**Table 4**). Good’s coverage estimates showed that all groups had more than 99% bacterial species. The salinity significantly affected the Sobs, Chao1, ACE, Shannon, and Simpson indices, while the protein source significantly affected the Chao1 and Simpson indices of the intestinal microbiota in *L. vannamei*. Besides, the interaction of protein source and salinity significantly affected the Sobs, Chao1, ACE, and Simpson indices of the intestinal microbiota (*P*< 0.05). The analysis of beta diversity by PCoA analysis showed that both the samples in the FM and the CAP groups had obvious separation under different salinity conditions, and the two PCoA axes showed 63.60% variation among the groups (**Figure 6**).

**Table 4:** Effects of protein sources and salinity on microflora diversity in the intestine of *L. vannamei.*

Items		Good’s coverage	Sobs	Chao1	ACE	Shannon	Simpson
Protein source	Salinity/‰					
**FM**	**15**	0.99 ± 0.00	555.00 ± 64.35^a^	722.04 ± 13.50^Bb^	635.19 ± 11.89^a^	3.97 ± 0.09^a^	0.75 ± 0.10^Aa^
	**30**	0.99 ± 0.00	525.00 ± 7.07^a^	588.28 ± 9.68^a^	610.46 ± 16.66^Aa^	4.29 ± 0.04^ab^	0.82 ± 0.02^b^
	**45**	0.99 ± 0.00	738.00 ± 31.95^Bb^	798.01 ± 20.33^Bc^	774.83 ± 8.49^b^	4.89 ± 0.10^b^	0.91 ± 0.03^b^
**CAP**	**15**	0.99 ± 0.00	562.00 ± 14.73	612.40 ± 2.29^Aa^	636.98 ± 13.29^a^	4.27 ± 0.42	0.88 ± 0.05^B^
	**30**	0.99 ± 0.00	528.33 ± 36.90	619.34 ± 25.69^a^	648.59 ± 11.87^Ba^	4.32 ± 0.29	0.88 ± 0.03
	**45**	0.99 ± 0.00	574.33 ± 70.69^A^	702.42 ± 7.89^Ab^	731.50 ± 3.34^b^	4.58 ± 0.13	0.89 ± 0.03
***P*-value of two-way ANOVA**							
**Salinity**		0.9255	0.0056	<0.0001	<0.0001	0.0024	0.0009
**Protein source**		0.0974	0.0844	<0.0001	0.8713	0.9332	0.0052
**Interaction**		0.3910	0.0348	<0.0001	0.0031	0.1219	0.0021

Values with different capital letter superscripts indicated significant differences between different protein sources at the same salinity (p<0.05). Values with different small letter superscripts indicated significant difference among different salinities when the protein source was the same (p<0.05).

#### 3.6.2 Comparison of the intestinal microbiota composition

**Figure 7:**
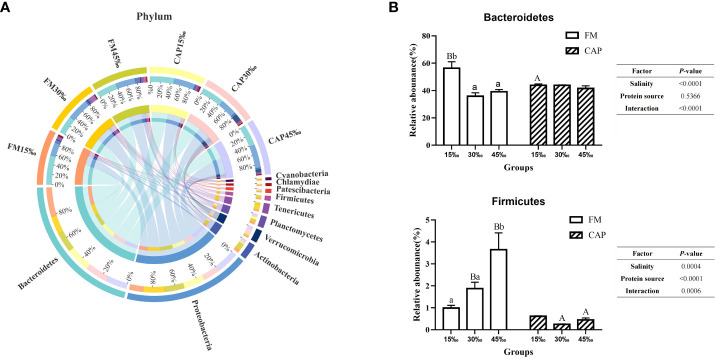
Effects of protein sources and salinity on the structure and composition of intestinal microbiota community in L. vannamei at phylum level. **(A)** Mean abundance indifferent groups. One side of the graph is the grouping information, and the other side is the species information. The lines on both sides represent corresponding relationship pairs. The thicker the lines, the greater the abundance value. **(B)** Relative abundance with significant differences in phylum levels. Under the same salinity, values with different capital letter superscripts mean significant difference among different protein source (*P*<0.05); under the protein sources, values with different small letter superscripts mean significant difference between different salinity (*P<* 0.05).

As shown in **Figure 7A**, the top 10 intestinal bacterial phyla of *L. vannamei* sorted from high to low were Bacteroidetes, Proteobacteria, Actinobacteria, Verrucomicrobia, Planctomycetes, Tenericutes, Firmicutes, Patescibacteria, Chlamydiae, and Cyanobacteria. As shown in **Figure 7B**, the relative abundances of Bacteroidetes in the FM group both at 30‰ and 45‰ salinities were significantly lower than that at 15‰ salinity (*P<* 0.05), while there was no significant difference in those in the CAP group at different salinities (*P* > 0.05). At 15‰ salinity, the relative abundance of Bacteroidetes in the CAP group was significantly lower than that in the FM group. The salinity but not the protein source significantly affected the relative abundance of Bacteroidetes. However, the interaction of salinity and protein source had a significant effect on the relative abundance of Bacteroidetes. The relative abundance of Firmicutes in the FM group at 45‰ salinity was significantly higher than those at 15‰ and 30‰ salinities (*P<* 0.05), while there was no significant difference in the CAP group at different salinities (*P* > 0.05). The salinity, protein sources, and their interaction had a significant effect on the relative abundance of Firmicutes (*P*< 0.05).

**Figure 8:**
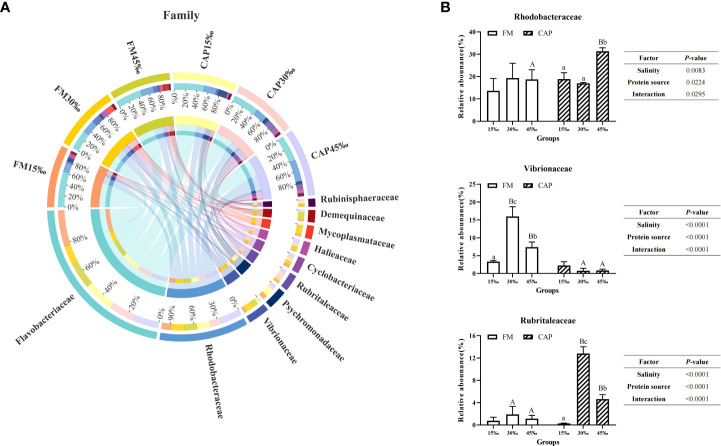
Effects of protein sources and salinity on the structure and composition of intestinal microbiota community in L. vannamei at family level. **(A)** Mean abundance indifferent groups. One side of the graph is the grouping information, and the other side is the species information. The lines on both sides represent corresponding relationship pairs. The thicker the lines, the greater the abundance value. **(B)** Relative abundance with significant differences in family levels. Under the same salinity, values with different capital letter superscripts mean significant difference among different protein source (*P*<0.05); under the protein sources, values with different small letter superscripts mean significant difference between different salinity (*P<* 0.05).

At the family level (**Figure 8A**), the prevalent microbial communities in the intestine of *L. vannamei* consisted of Flavobacteriaceae, Rhodobacteraceae, Vibrionaceae, Psychromonadaceae, and Rubritaleaceae. As shown in **Figure 8B**, the change in salinity had no significant effect on the relative abundance of Rhodobacteraceae and Rubrialeaceae in the FM group and the relative abundance of Vibrionaceae in the CAP group. In the CAP group, the relative abundance of Rhodobacteraceae at 45‰ salinity was not only significantly higher than those at 15‰ and 30‰ but also significantly higher than that in the FM group at the same salinity (*P<* 0.05). The relative abundances of Vibrionaceae were significantly different among the FM groups at the three different salinities, with the highest level at 30‰ salinity, and the relative abundances of Vibrionaceae in the CAP group at 30‰ and 45‰ salinities were significantly lower than that in the FM group at the same salinity. In the CAP group, the relative abundances of Rubrialeaceae were significantly different at the three different salinities. When compared with the FM group, the relative abundances of Rubrialeaceae were significantly increased in the CAP group at 30‰ and 45‰ salinities (*P*< 0.05). The salinity, protein sources, and their interaction significantly affected the relative abundances of Rhodobacteraceae, Vibrionaceae, and Rubrialeaceae (*P<* 0.05).

**Figure 9:**
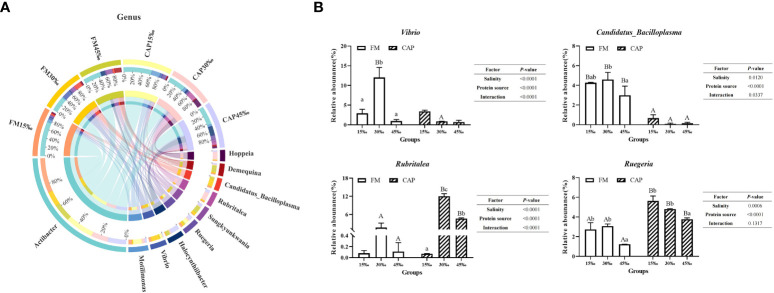
Effects of protein sources and salinity on the structure and composition of intestinal microbiota community in *L. vannamei* at genus level. **(A)** Mean abundance indifferent groups. One side of the graph is the grouping information, and the other side is the species information. The lines on both sides represent corresponding relationship pairs. The thicker the lines, the greater the abundance value. **(B)** Relative abundance with significant differences in genus levels. Under the same salinity, values with different capital letter superscripts mean significant difference among different protein source (*P*<0.05); under the protein sources, values with different small letter superscripts mean significant difference between different salinity (*P<* 0.05).

At the genus level (**Figure 9A**), *Actibacter* was the species with the highest abundance, followed by *Motilimonas*, *Vibrio*, *Halocynthiibacter*, and *Ruegeria*. As shown in **Figure 9B**, the change in salinity had a significant effect on the relative abundance of *Vibrio* and *Candidatus_Bacilloplasma* in the FM group but not in the CAP group. The relative abundance of *Vibrio* in the FM group at 30‰ salinity was significantly higher than those at 15‰ and 45‰ salinities. The relative abundance of *Vibrio* at 30‰ salinity in the CAP group was significantly lower than that in the FM group. With the increase in salinity, the relative abundance of *Candidatus_Bacilloplasma* in the FM group increased first and then decreased, with the only insignificant difference between 15‰ and 30‰ salinities. At all the three studied salinities, the relative abundance of *Candidatus_Bacilloplasma* in the CAP group was significantly lower than that in the FM group. The change in salinity had a significant effect on the relative abundance of *Rubritalea* in the CAP group but not in the FM group. The relative abundances of *Rubritalea* were significantly different from each other in the CAP group at all the three studied salinities, with the highest level at 30‰ salinity. At 30‰ and 45‰ salinities, the relative abundances of *Rubritalea* in the CAP group were significantly higher than that in the FM group (*P<* 0.05). The relative abundances of *Ruegeria* both in the FM and the CAP groups at 45‰ salinity were significantly lower than those at the 15‰ and 30‰ salinities. When compared with the FM group, the relative abundances of *Ruegeria* were significantly increased in the CAP group at all the three studied salinities (*P<* 0.05). Besides, the salinity and protein sources significantly affected the relative abundances of *Vibrio*, *Candidatus_Bacilloplasma*, *Rubritalea*, and *Ruegeria* (*P<* 0.05), while the interaction of the salinity and protein sources had a significant effect on the relative abundances of *Vibrio*, *Candidatus_Bacilloplasma*, and *Rubritalea* but not on that of *Ruegeria.*

#### 3.6.3 Functional prediction of the intestinal microbial community

**Figure 10:**
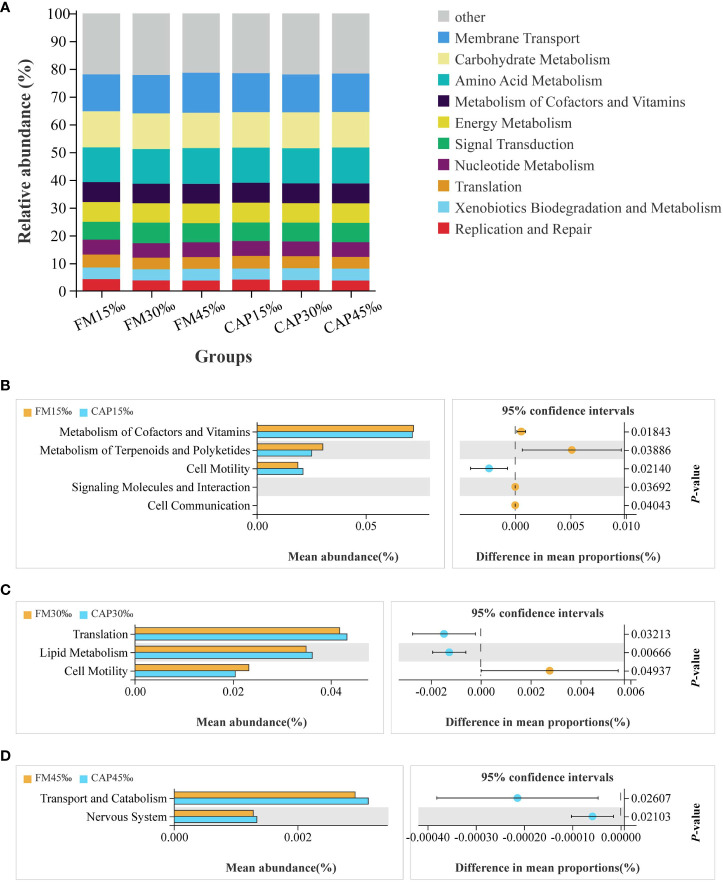
Functional prediction of protein sources and salinity in intestinal microbial community. **(A) **Relative abundances of the top 10 predicted functions. **(B–D)** Welch’s t-tests of the significantly different functions at level 2.

Changes in the presumptive functions of intestinal microflora were examined using Tax4fun software to predict the metagenomes. As shown in **Figure 10A**, the top 10 predicted functions had the following relative abundances: membrane transport (13.28–14.40%), carbohydrate metabolism (12.71–12.99%), amino acid metabolism (12.48–12.95%), metabolism of cofactors and vitamins (7.03–7.20%), energy metabolism (6.98–7.14%), signal transduction (6.43–7.39%), nucleotide metabolism (5.23–5.38%), translation (4.18–4.65%), xenobiotic biodegradation and metabolism (3.96–4.30%), and replication and repair (3.74–4.24%).

The results of Welch’s t-test showed that there were several predicted pathways for significant enrichment in the microbial community with KEGG level 2 at different salinities of the same protein source (*P*< 0.05). As shown in **Figure 10B**, the enriched functions related to cell motility were significantly increased, while the functions of metabolism of cofactors and vitamins, metabolism of terpenoids and polyketides, signalling molecules and interaction, and cell communication were significantly decreased in the CAP group when compared with the FM group at 15 ‰ salinity. At 30 ‰ salinity (**Figure 10C**), the functions of translation and lipid metabolism were significantly increased, while the function of cell motility was significantly decreased in the CAP group when compared with that in the FM group. Comparison between the CAP and FM groups at 45‰ salinity indicated that the functions of transport, catabolism, and the nervous system were significantly increased in the CAP group (**Figure 10D**).

## 4 Discussion

There is a great demand for fishmeal in the culture of *L. vannamei*. At present, the substitution of fishmeal has attracted much attention in *L. vannamei* culture due to its shortage (10). CAP has been used in aquaculture to improve the growth performance, feed utilization, and immune response of *L. vannamei (* 27). Salinity is a crucial environmental factor for *L. vannamei* culture and has a great impact on the utilization of protein (44). In this study, the growth, disease resistance, intestinal digestive capacity, immunity, and microbiota structure of *L. vannamei* fed on CAP at different salinities were investigated to evaluate the application value of CAP in shrimp culture.

As a high-quality SCP, CAP has been used for several aquaculture species with various beneficial effects. In *A. schlegelii*, CAP can replace fishmeal in the diet up to 58.20% without adverse effects on growth performance, antioxidation, and digestive enzyme activity (26). In *M. salmoides*, the replacement of fishmeal with CAP did not affect its growth performance and whole-body composition but increased the digestive capacity and antioxidant index. The optimal CAP replacement level was 49.80% with a maximum WGR of juvenile *M. salmoides (* 31). Lu et al. found that there is no negative effect on the growth performance and liver health of *M. salmoides* when the level of CAP replacing fishmeal is less than 50%, and excessive CAP inclusion may damage liver health (29). CAP supplementation in practical diets has a beneficial effect on the growth performance of *C. carpio* var. *Jian (* 28) and *O. niloticus (* 27). In addition, the antioxidant capacity of *C. carpio* var. *Jian* was increased and the whole-body energy homeostasis of *O. niloticus* could be regulated through the AMPK signalling pathway. In *L. vannamei*, CAP could substitute 30% of fishmeal in a diet containing 560 g/kg of fishmeal without adverse effects on growth, intestinal histology, and immunity (27). Consistent with the previous study performed in *L. vannamei*, our study also found that the growth performance, digestive capacity, and immunity of *L. vannamei* could be improved by dietary CAP. The improved growth of *L. vannamei* was evident since the WGR and SGR of *L. vannamei* in the CAP group were significantly higher than those in the FM group at 30‰ and 45‰ salinities. Moreover, the SR, WGR, and SGR of *L. vannamei* were not negatively affected by dietary CAP at all the studied salinities. The digestive capacity of *L. vannamei* was improved since both the activities and the expressions of the digestive enzymes were significantly increased in the CAP group when compared with those in the FM group. Furthermore, dietary CAP significantly increased the SR of *L. vannamei* after a pathogen challenge, which indicated that the immunity of *L. vannamei* was improved by dietary CAP. However, the FCR of *L. vannamei* in the CAP group was significantly higher than that in the FM group, while the comparison of PER between the CAP and the FM groups was opposite at 30‰ and 45‰ salinities. The increased FCR and decreased PER suggested that CAP may not be perfect. Nonetheless, the possibility of using CAP as a safe and effective alternative protein source in aquatic feed is beyond doubt.

The intestinal histology and digestive capacity of fish and shrimp fed on CAP have been investigated before (27, 31). However, there were no reports on the effect of dietary CAP on aquatic animals from the aspect of intestinal microbiota structures. In recent years, the sequencing technology of intestinal microbiota has received extensive attention. Studies have shown that the intestinal microbiota is closely linked to digestion, immunity, metabolism, and the overall health of the host (45, 46). Based on this, the intestinal microbiota structure of *L. vannamei* fed on CAP was compared with that of *L. vannamei* fed on FM. Alpha diversity analysis showed that the diversity and abundance of the intestinal microbiota of *L. vannamei* in the CAP group were significantly different from those in the FM group, suggesting that dietary CAP would change the diversity of intestinal microbiota. Bacteroidetes and Proteobacteria were considered the dominant phyla in the intestine of fish and shrimp (47–50). In this study, the two phyla were the first and second dominant phyla both in the CAP and the FM groups, and there was no significant difference in their relative abundance between the CAP and the FM groups, which further proved that there was no harm in the intestinal microbiota diversity of *L. vannamei* when fed on CAP. An interesting discovery was that the beneficial bacteria significantly increased while the harmful bacteria significantly decreased in the intestine of *L. vannamei* at the phylum, family, and genus levels. The effect was more obvious at the genus level. *Vibrio* is the most common core bacterial group in the intestine of crustaceans (51). Several members in the *Vibrio* genus are conditionally pathogenic bacteria, which seriously affects the survival and health of shrimp, such as *V. parahaemolyticus (* 52), *Vibrio harveyi (* 53), and *Vibrio alginolyticus (* 54). White faeces syndrome (WFS) is a severe disease and has drawn wide attention in shrimp culture. Huang et al. found that *Vibrio* and *Candidatus_Bacilloplasma* were the two overrepresented genera in shrimp with WFS (55). In this study, the relative abundance of *Vibrio* genus in the CAP group was significantly lower than that in the FM group at a salinity of 30‰, and the relative abundance of *C._Bacilloplasma* was significantly increased in the CAP group at 30‰ and 45‰ salinities. On the contrary, the relative abundances of *Rubritalea* and *Ruegeria* were significantly increased by dietary CAP. *Rubritalea* is a genus in the phylum Verrucomicrobia that could degrade the excess mucin produced by the inner wall of the intestine and was helpful for normal growth (56). *Ruegeria* is a global gram-negative marine bacterium that can co-exist with unicellular eukaryotes and inhibit many marine pathogens (57, 58). It could be speculated that since the change in the intestinal microbiota structure of *L. vannamei* was associated with increased disease resistance of *L. vannamei* in the CAP group when compared with the FM group, it may make a valuable contribution to increasing disease resistance. Additionally, the predictive function of intestinal microflora also revealed that metabolism-related functions account for most of the top 10 functions and are closely related to the promotion of *L. vannamei* growth. However, further study is needed to confirm this speculation.

Several indices can be used to evaluate the immunity of shrimp, including the activities of SOD, LGBP, antibacterial peptide (AMP), and so on. SOD is an important antioxidant enzyme that provided the first line of ROS elimination from cells (59). As a pattern recognition protein (PRP), LGBP is involved in the activation of shrimp immunity by recognizing LPS and β-1,3-glucan from gram-negative bacteria (60). The prophenoloxidase (proPO) system is an enzyme cascade system that is activated upon recognition of pathogens by PRPs like LGBP and plays an important role in the innate immunity of shrimp (61). Crustins, ALFs, and PEN are the three important kinds of AMPs that contribute to the antibacterial capabilities of shrimp as efficient effectors (62). Lysozyme plays an important role in the shrimp’s immune defence by destroying peptidoglycan support, resulting in bacterial splitting under osmotic pressure within the bacteria (63). In this study, the relative expression levels of immune genes, including SOD, LGBP, PPO, PO, CRU, ALF, PEN, and LYZ, in the CAP group were higher than those in the FM group, which indicated that the intestinal immunity of *L. vannamei* could be enhanced by dietary CAP. The results were consistent with those of the intestinal microbiota analysis.

Since *L. vannamei* have an open hemolymph circulation system, they adjust their osmotic pressure effectively to adapt to the changes in external salinity (15, 16). Osmotic adjustment is an energy-dependent process. When facing changes in external salinity, providing adequate energy by manipulating the diet is an effective means of improving shrimp’s survival abilities (21, 64). Li et al. found that the digestive and immune regulatory abilities of *L. vannamei* remained at a high level, and the expression level of antioxidant-related genes is also significant during salinity stress (44). Obviously, all the regulatory processes in *L. vannamei* required energy consumption. If the energy taken in from the external environment is not enough, *L. vannamei* will use their own body energy, resulting in a rapid reduction in growth. In this study, the growth performance of *L. vannamei* at 30‰ salinity was much better than that at 15‰ or 45‰ salinities, whether the protein source in the feed was FM or CAP. This was probably because the extra energy *L. vannamei* obtained from the feed could be used for growth at a proper salinity rather than coping with the pressure caused by low or high salinities. The activities and expression of intestinal digestive enzymes in *L. vannamei* at 45‰ salinity were significantly higher than those at 30‰ salinity, which further implied that *L. vannamei* need to take in more energy from the external environment in response to salinity stress. What cannot be ignored is that the FCR and PER of *L. vannamei* in the FM group were not obviously affected by the salinity change but they were significantly affected by the increase in salinity. Besides, the SR of *L. vannamei* after a pathogen challenge was only significantly affected by salinity change in the CAP group but not in the FM group. Differently, most of the expressions of immune genes were significantly increased with the increase in salinity both in the FM and the CAP groups. Furthermore, the changes in several main intestinal flora species at different levels of *L. vannamei* affected by salinity increase in the CAP group were significantly different from those in the FM group. All these results indicated that salinity change variably affected *L. vannamei* in several aspects when the protein sources were different.

Finally, our study found that the interaction of salinity and protein source significantly affected *L. vannamei* in most of the aspects studied, including growth performance, activities and expression of digestive enzymes, expression of immune genes, and abundance of special microbiota species at the phyla, family, and genus levels. The results suggest that the change in salinity had a significant impact on the effect of dietary CAP on *L. vannamei*, which could provide a theoretical reference for the practical application of CAP in aquatic feeds.

In conclusion, on the one hand, dietary CAP could effectively improve growth performance, disease resistance, intestinal digestive capacity, immunity, and microbial structure but not feed utilization in *L. vannamei* under the same salinity condition. On the other hand, the change in salinity had much more obvious effects on *L. vannamei* fed on CAP than the control in the aspects of growth performance, disease resistance, intestinal digestive capacity, immunity, and microbiota structure. In any case, CAP could be used as a safe and effective alternative protein source in shrimp feeds.

## Data availability statement

Publicly available datasets were analyzed in this study. This data can be found here: NCBI [accession: PRJNA870236].

## Ethics statement

This study was reviewed and approved by Guangdong Ocean University.

## Author contributions

JC: Conceptualization, Investigation, Formal analysis, Writing-Original Draft. HW, HY, NH, FZ, CL, LS and BT: Methodology, Resources. SZ: Review and Editing, Supervision, Project administration, Funding acquisition. All authors contributed to the article and approved the submitted version.

## Funding

This work was supported by the National Key R&D Program of China (Grant No. 2019YFD0900200), National Natural Science Foundation of China (Grant No. 32072988), General Program of Natural Science Foundation of Guangdong Province, China (Grant No. 2020A1515010319) and Guangdong Postgraduate Education Innovation Project (Grant No. 202266).

## Acknowledgments

We thank Beijing Shoulang bio-technology Co., Ltd. for providing *Clostridium autoethanogenum* protein and Guangzhou Genedenovo Biotechnology Co., Ltd (Guangdong, China) for the services of sequencing and bioinformatics analysis (http://www.omicsmart.com).

## Conflict of interest

Author FZ and CL were employed by Beijing Shoulang Bio-Technology Co., Ltd., Beijing, China.

The remaining authors declare that the research was conducted in the absence of any commercial or financial relationships that could be construed as a potential conflict of interest.

## Publisher’s note

All claims expressed in this article are solely those of the authors and do not necessarily represent those of their affiliated organizations, or those of the publisher, the editors and the reviewers. Any product that may be evaluated in this article, or claim that may be made by its manufacturer, is not guaranteed or endorsed by the publisher.

## References

[1] Hicks CC Cohen PJ Graham NAJ Nash KL Allison EH D’Lima C. Harnessing global fisheries to tackle micronutrient deficiencies. Nature (2019) 574:95–8. doi: 10.1038/s41586-019-1592-6 31554969

[2] FAO. The state of world fisheries and aquaculture: Contributing to food security and nutrition for all. Rome: FAO (2016). 200 p. doi: 10.18356/8e4e0ebf-en

[3] FAO. The state of world fisheries and aquaculture 2020. Rome: FAO (2020). 206 p. doi: 10.4060/ca9229en

[4] Council NR. Nutrient requirements of fish and shrimp. Washington, D.C.: National Academies Press (2011). doi: 10.17226/13039

[5] Naylor RL Hardy RW Buschmann AH Bush SR Cao L Klinger DH. A 20-year retrospective review of global aquaculture. Nature (2021) 591:551–63. doi: 10.1038/s41586-021-03308-6 33762770

[6] Dulvy NK Pacoureau N Rigby CL Pollom RA Jabado RW Ebert DA. Overfishing drives over one-third of all sharks and rays toward a global extinction crisis. Curr Biol (2021) 31:4773–4787.e8. doi: 10.1016/J.CUB.2021.08.062 34492229

[7] Henry M Gasco L Piccolo G Fountoulaki E. Review on the use of insects in the diet of farmed fish: Past and future. Anim Feed Sci Technol (2015) 203:1–22. doi: 10.1016/J.ANIFEEDSCI.2015.03.001

[8] FAO. FAO yearbook. fishery and aquaculture statistics 2019/FAO annuaire. statistiques des pêches et de l’aquaculture 2019/FAO anuario. estadísticas de pesca y acuicultura 2019. Rome: FAO (2021). 82 p. doi: 10.4060/cb7874t

[9] Kureshy N Allen Davis D. Protein requirement for maintenance and maximum weight gain for the pacific white shrimp. Litopenaeus vannamei Aquacult (2002) 204:125–43. doi: 10.1016/S0044-8486(01)00649-4

[10] Ayisi CL Hua X Apraku A Afriyie G Kyei BA. Recent studies toward the development of practical diets for shrimp and their nutritional requirements. Hayati (2017) 24:109–17. doi: 10.1016/J.HJB.2017.09.004

[11] Zhang WQ. Introduction to *Litopenaeus vannamei*: One of the most important breeding species in the world. Mar Sci (1990) 3:69–72. doi: 10.1016/j.aqrep.2020.100423

[12] Zhao YC Li YQ Sun ZP Wang SS Fu RJ Zhang SL. Effects of high-salinity domestication gradient, speed, and mode on weight gain, activity, and survival rate of *Litopenaeus vannamei* post larvae. Prog Fish Sci (2018) 39:119–25. doi: 10.19663/j.issn2095-9869.20171010002

[13] Ray AJ Lotz JM. Comparing salinities of 10, 20, and 30‰ in intensive, commercial scale biofloc shrimp (*Litopenaeus vannamei*) production systems. Aquaculture (2017) 476:29–36. doi: 10.1016/J.AQUACULTURE.2017.03.047

[14] Chen K Li E Xu C Wang X Li H Qin JG. Growth and metabolomic responses of pacific white shrimp (*Litopenaeus vannamei*) to different dietary fatty acid sources and salinity levels. Aquaculture (2019) 499:329–40. doi: 10.1016/J.AQUACULTURE.2018.09.056

[15] Ponce-Palafox JT Pavia ÁA Mendoza López DG Arredondo-Figueroa JL Lango-Reynoso F Castañeda-Chávez M del R. Response surface analysis of temperature-salinity interaction effects on water quality, growth and survival of shrimp *Penaeus vannamei* post larvae raised in biofloc intensive nursery production. Aquaculture (2019) 503:312–21. doi: 10.1016/J.AQUACULTURE.2019.01.020

[16] Jannathulla R Syama Dayal J Chitra V Ambasankar K Muralidhar M. Growth and carcass mineralisation of pacific white leg shrimp *Penaeus vannamei* Boone 1931 in response to water salinity. Indian J Fish (2017) 64:22–7. doi: 10.21077/IJF.2017.64.2.59404-04

[17] Gao WH Chi SY Tan BP Liu HY Dong XH Yang QH. Construction of the hepatopancreas subtractive cDNA library of *Litopenaeus vannamei* induced by hyposmotic stress and ESTs analysis. J Guangdong Ocean Univ (2012) 6:1–9. doi: 10.3969/j.issn.1673-9159.2012.06.003

[18] Guo H Tan CT You LY Shen YC Lu ZC Zhu CH. Effects of nitrite stress on gene expression of antioxidant enzymes, heat shock protein and cathepsin b in hepatopancreas of. Litopenaeus vannamei J Guangdong Ocean Univ (2017) 37:117–22. doi: 10.3969/j.issn.1673-9159.2017.03.018

[19] Valencia-Castañeda G Frías-Espericueta MG Vanegas-Pérez RC Chávez-Sánchez MC Páez-Osuna F. Physiological changes in the hemolymph of juvenile shrimp *Litopenaeus vannamei* to sublethal nitrite and nitrate stress in low-salinity waters. Environ Toxicol Pharmacol (2020) 80:103–472. doi: 10.1016/J.ETAP.2020.103472 32822850

[20] Abrori M Soegianto A Winarni D. Survival, osmoregulatory and hemocyte changes in *Litopenaeus vannamei* postlarvae acclimated to different intervals of salinity reduction. Aquac Rep (2022) 25:101222. doi: 10.1016/J.AQREP.2022.101222

[21] Wang XD Li EC Wang SF Qin JG Chen XF Lai QM. Protein-sparing effect of carbohydrate in the diet of white shrimp *Litopenaeus vannamei* at low salinity. Aquac Nutr (2015) 21:904–12. doi: 10.1111/anu.12221

[22] Huang K Wang W Lu J. Protein requirements in compounded diets for *Penaeus vannamei* juveniles. J Fish Sci China (2003) 10:318–24. doi: 10.3321/j.issn:1005-8737.2003.04.011

[23] Sui L Ma G Deng Y. Effect of dietary protein level and salinity on growth, survival, enzymatic activities and amino-acid composition of the white shrimp *Litopenaeus vannamei* (Boone, 1931) juveniles. Crustaceana (2015) 88:82–95. doi: 10.1163/15685403-00003390

[24] Karlsen F Skov PV. Review – potentials and limitations of utilising brewer’s spent grain as a protein source in aquaculture feeds. J Clean Prod (2022) 357:131986. doi: 10.1016/j.jclepro.2022.131986

[25] Nasseri AT Rasoul-Amini S Morowvat MH Ghasemi Y. Single cell protein: Production and process. Am J Food Technol (2011) 6:103–16. doi: 10.3923/AJFT.2011.103.116

[26] Chen Y Sagada G Xu B Chao W Zou F Ng WK. Partial replacement of fishmeal with *Clostridium autoethanogenum* single-cell protein in the diet for juvenile black sea bream (*Acanthopagrus schlegelii*). Aquac Res (2020) 51:1000–11. doi: 10.1111/ARE.14446

[27] Jiang X Yao W Yang H Tan S Leng X Li X. Dietary effects of *Clostridium autoethanogenum* protein substituting fish meal on growth, intestinal histology and immunity of pacific white shrimp (*Litopenaeus vannamei*) based on transcriptome analysis. Fish Shellfish Immunol (2021) 119:635–44. doi: 10.1016/J.FSI.2021.10.005 34740770

[28] Li M Liang H Xie J Chao W Zou F Ge X. Diet supplemented with a novel *Clostridium autoethanogenum* protein have a positive effect on the growth performance, antioxidant status and immunity in juvenile jian carp (*Cyprinus carpio* var. *Jian*). Aquac Rep (2021) 19:100572. doi: 10.1016/J.AQREP.2020.100572

[29] Lu Q Xi L Liu Y Gong Y Su J Han D. Effects of dietary inclusion of *Clostridium autoethanogenum* protein on the growth performance and liver health of largemouth bass (*Micropterus salmoides*). Front Mar Sci (2021) 8:764964/BIBTEX. doi: 10.3389/FMARS.2021.764964/BIBTEX

[30] Maulu S Liang H Ge X Yu H Huang D Ke J. Effect of dietary *Clostridium autoethanogenum* protein on growth, body composition, plasma parameters and hepatic genes expression related to growth and AMPK/TOR/PI3K signaling pathway of the genetically improved farmed tilapia (*GIFT: Oreochromis niloticus*) juveniles. Anim Feed Sci Technol (2021) 276:114914. doi: 10.1016/J.ANIFEEDSCI.2021.114914

[31] Zhu S Gao W Wen Z Chi S Shi Y Hu W. Partial substitution of fish meal by *Clostridium autoethanogenum* protein in the diets of juvenile largemouth bass (*Micropterus salmoides*). Aquac Rep (2022) 22:100938. doi: 10.1016/J.AQREP.2021.100938

[32] Yao W Yang P Zhang X Xu X Zhang C Li X. Effects of replacing dietary fish meal with *Clostridium autoethanogenum* protein on growth and flesh quality of pacific white shrimp (*Litopenaeus vannamei*). Aquaculture (2022) 549:737770. doi: 10.1016/J.AQUACULTURE.2021.737770

[33] Zhu C DONG S-L Wang F. The interaction of salinity and Na/K ratio in seawater on growth, nutrient retention and food conversion of juvenile *Litopenaeus vannamei* . J Shellfish Res (2009) 25:107–12. doi: 10.2983/0730-8000(2006)25[107:TIOSAK]2.0.CO;2

[34] Camacho-Jiménez L Díaz F Sánchez-Castrejón E Ponce-Rivas E. Effects of the recombinant crustacean hyperglycemic hormones rCHH-B1 and rCHH-B2 on the osmo-ionic regulation of the shrimp *Litopenaeus vannamei* exposed to acute salinity stress. J Comp Physiol B (2018) 188:565–79. doi: 10.1007/S00360-018-1151-8 29582134

[35] Shi L Chan S Li C Zhang S. Identification and characterization of a laccase from *Litopenaeus vannamei* involved in anti-bacterial host defense. Fish Shellfish Immunol (2017) 66:1–10. doi: 10.1016/J.FSI.2017.04.026 28476665

[36] Yao Y Shi L Xiao W Guo S Liu S Li H. Phenylalanine hydroxylase (PAH) plays a positive role during WSSV and *Vibrio parahaemolyticus* infection in *Litopenaeus vannamei* . Fish Shellfish Immunol (2022) 120:515–25. doi: 10.1016/J.FSI.2021.12.028 34952194

[37] Livak KJ Schmittgen TD. Analysis of relative gene expression data using real-time quantitative PCR and the 2(-delta C(T)) method. Methods (2001) 25:402–8. doi: 10.1006/METH.2001.1262 11846609

[38] Edgar RC. UPARSE: highly accurate OTU sequences from microbial amplicon reads. Nat Methods (2013) 10:996–8. doi: 10.1038/NMETH.2604 23955772

[39] Wang Q Garrity GM Tiedje JM Cole JR. Naive Bayesian classifier for rapid assignment of rRNA sequences into the new bacterial taxonomy. Appl Environ Microbiol (2007) 73:5261–7. doi: 10.1128/AEM.00062-07 PMC1950982 17586664

[40] Pruesse E Quast C Knittel K Fuchs BM Ludwig W Peplies J. SILVA: A comprehensive online resource for quality checked and aligned ribosomal RNA sequence data compatible with ARB. Nucleic Acids Res (2007) 35:7188–96. doi: 10.1093/NAR/GKM864 PMC2175337 17947321

[41] Caporaso JG Kuczynski J Stombaugh J Bittinger K Bushman FD Costello EK. QIIME allows analysis of high-throughput community sequencing data. Nat Methods (2010) 7:335–6. doi: 10.1038/NMETH.F.303 PMC3156573 20383131

[42] Aßhauer KP Wemheuer B Daniel R Meinicke P. Tax4Fun: predicting functional profiles from metagenomic 16S rRNA data. Bioinformatics (2015) 31:2882–4. doi: 10.1093/BIOINFORMATICS/BTV287 PMC4547618 25957349

[43] Stevens MHH Wagner H. Vegan: community ecology package. r package version 1.17-4(2010). Available at: http://CRAN.R-project.org/package=vegan.

[44] Li E Wang X Chen K Xu C Qin JG Chen L. Physiological change and nutritional requirement of pacific white shrimp *Litopenaeus vannamei* at low salinity. Rev Aquac (2017) 9:57–75. doi: 10.1111/RAQ.12104

[45] Baldo L Riera JL Tooming-Klunderud A Albà MM Salzburger W. Gut microbiota dynamics during dietary shift in eastern African cichlid fishes. PloS One (2015) 10:e0127462. doi: 10.1371/JOURNAL.PONE.0127462 25978452 PMC4433246

[46] Yukgehnaish K Kumar P Sivachandran P Marimuthu K Arshad A Paray BA. Gut microbiota metagenomics in aquaculture: Factors influencing gut microbiome and its physiological role in fish. Rev Aquac (2020) 12:1903–27. doi: 10.1111/RAQ.12416

[47] Rungrassamee W Klanchui A Maibunkaew S Chaiyapechara S Jiravanichpaisal P Karoonuthaisiri N. Characterization of intestinal bacteria in wild and domesticated adult black tiger shrimp (*Penaeus monodon*). PloS One (2014) 9:e91853. doi: 10.1371/JOURNAL.PONE.0091853 24618668 PMC3950284

[48] Xiong J Wang K Wu J Qiuqian L Yang K Qian Y. Changes in intestinal bacterial communities are closely associated with shrimp disease severity. Appl Microbiol Biotechnol (2015) 99:6911–9. doi: 10.1007/S00253-015-6632-Z 25947250

[49] Dehler CE Secombes CJ Martin SAM. Environmental and physiological factors shape the gut microbiota of Atlantic salmon parr (*Salmo salar l.*). Aquaculture (2017) 467:149–57. doi: 10.1016/J.AQUACULTURE.2016.07.017 PMC5142738 28111483

[50] Amoah K Huang QC Tan BP Zhang S Chi SY Yang QH. Dietary supplementation of probiotic bacillus coagulans ATCC 7050, improves the growth performance, intestinal morphology, microflora, immune response, and disease confrontation of pacific white shrimp, *Litopenaeus vannamei* . Fish Shellfish Immunol (2019) 87:796–808. doi: 10.1016/J.FSI.2019.02.029 30790661

[51] Wang R Guo Z Tang Y Kuang J Duan Y Lin H. Effects on development and microbial community of shrimp *Litopenaeus vannamei* larvae with probiotics treatment. AMB Express (2020) 10:109. doi: 10.1186/s13568-020-01041-3 32504358 PMC7275112

[52] Nguyen TV Alfaro A BB A Leon JAR Sonnenholzner S. Metabolic responses of penaeid shrimp to acute hepatopancreatic necrosis disease caused by *Vibrio parahaemolyticus* . Aquaculture (2021) 533:736174. doi: 10.1016/j.aquaculture.2020.736174

[53] Liu H Guo S Wang R He Y Shi Q Song Z. Pathogen of *Vibrio harveyi* infection and c-type lectin proteins in white leg shrimp (*Litopenaeus vannamei*). Fish Shellfish Immunol (2021) 119:554–62. doi: 10.1016/j.fsi.2021.10.040 34718124

[54] Liao G Wu Q Mo B Zhou J Li J Zou J. Intestinal morphology and microflora to *Vibrio alginolyticus* in pacific white shrimp (*Litopenaeus vannamei*). Fish Shellfish Immunol (2022) 121:437–45. doi: 10.1016/J.FSI.2022.01.026 35065276

[55] Huang Z Zeng S Xiong J Hou D Zhou R Xing C. Microecological koch’s postulates reveal that intestinal microbiota dysbiosis contributes to shrimp white feces syndrome. Microbiome (2020) 8:32. doi: 10.1186/s40168-020-00802-3 32156316 PMC7065354

[56] Ito T Yoshiguchi K Ariesyady HD Okabe S. Identification and quantification of key microbial trophic groups of methanogenic glucose degradation in an anaerobic digester sludge. Bioresour Technol (2012) 123:599–607. doi: 10.1016/j.biortech.2012.07.108 22944494

[57] Bruhn JB Gram L Belas R. Production of antibacterial compounds and biofilm formation by *Roseobacter* species are influenced by culture conditions. Appl Environ Microbiol (2007) 73:442–50. doi: 10.1128/AEM.02238-06 PMC1796973 17098910

[58] Barreto-Curiel F Ramirez-Puebla ST Ringø E Escobar-Zepeda A Godoy-Lozano E Vazquez-Duhalt R. Effects of extruded aquafeed on growth performance and gut microbiome of juvenile *Totoaba macdonaldi* . Anim Feed Sci Technol (2018) 245:91–103. doi: 10.1016/J.ANIFEEDSCI.2018.09.002

[59] González-Ruiz R Granillo-Luna ON Peregrino-Uriarte AB Gómez-Jiménez S Yepiz-Plascencia G. Mitochondrial manganese superoxide dismutase from the shrimp *Litopenaeus vannamei*: Molecular characterization and effect of high temperature, hypoxia and reoxygenation on expression and enzyme activity. J Therm Biol (2020) 88:102519. doi: 10.1016/J.JTHERBIO.2020.102519 32125996

[60] Chen YY Chen JC Kuo YH Lin YC Chang YH Gong HY. Lipopolysaccharide and β-1,3-glucan-binding protein (LGBP) bind to seaweed polysaccharides and activate the prophenoloxidase system in white shrimp *Litopenaeus vannamei* . Dev Comp Immunol (2016) 55:144–51. doi: 10.1016/J.DCI.2015.10.023 26522339

[61] Amparyup P Sutthangkul J Charoensapsri W Tassanakajon A. Pattern recognition protein binds to lipopolysaccharide and β-1,3-glucan and activates shrimp prophenoloxidase system. J Biol Chem (2012) 287:10060–9. doi: 10.1074/JBC.M111.294744 PMC3322982 22235126

[62] Li C Wang S He J. The two NF-κB pathways regulating bacterial and WSSV infection of shrimp. Front Immunol (2019) 10:1785. doi: 10.3389/FIMMU.2019.01785 31417561 PMC6683665

[63] Cai S Zhang Y Wu F Wu R Yang S Li Y. Identification and functional characterization of a c-type lysozyme from *Fenneropenaeus penicillatus* . Fish Shellfish Immunol (2019) 88:161–9. doi: 10.1016/J.FSI.2019.02.043 30802628

[64] Chen K Li E Gan L Wang X Xu C Lin H. Growth and lipid metabolism of the pacific white shrimp *Litopenaeus vannamei* at different salinities. J Shellfish Res (2014) 33:825–32. doi: 10.2983/035.033.0317

